# Reputation-Aware Recruitment and Credible Reporting for Platform Utility in Mobile Crowd Sensing with Smart Devices in IoT

**DOI:** 10.3390/s18103305

**Published:** 2018-10-01

**Authors:** Waqas Ahmad, Shengling Wang, Ata Ullah, Sheharyar, Muhammad Yasir Shabir

**Affiliations:** 1 College of Information Science and Technology, Beijing Normal University, Beijing 100875, China; waqas@mail.bnu.edu.cn (W.A.); sheharyar@mail.bnu.edu.cn (S.); 2 Department of Computer Science, National University of Modern Languages, Islamabad 44000, Pakistan; Aullah@numl.edu.pk; 3 School of Computer and Communication Engineering, University of Science and Technology Beijing, Beijing 100083, China; 4 Department of CS & IT, University of Kotli, Azad Jammu and Kashmir, Kotli 10250, Pakistan; Yasir.shabir@uokajk.edu.pk

**Keywords:** Internet of Things (IoT), mobile crowd sensing (MCS), individual rationality, truthfulness, social welfare

## Abstract

The Internet of things (IoT) comprises a huge collection of electronic devices connected to the Internet to ensure the dependable exchange of sensing information. It involves mobile workers (MWs) who perform various activities to support enormous online services and applications. In mobile crowd sensing (MCS), a massive amount of sensing data is also generated by smart devices. Broadly, in the IoT, verifying the credibility and truthfulness of MWs’ sensing reports is needed for MWs to expect attractive rewards. MWs are recruited by paying monetary incentives that must be awarded according to the quality and quantity of the task. The main problem is that MWs may perform false reporting by sharing low-quality reported data to reduce the effort required. In the literature, false reporting is improved by hiring enough MWs for a task to evaluate the trustworthiness and acceptability of information by aggregating the submitted reports. However, it may not be possible due to budget constraints, or when malicious reporters are not identified and penalized properly. Recruitment is still not a refined process, which contributes to low sensing quality. This paper presents Reputation, Quality-aware Recruitment Platform (*RQRP*) to recruit MWs based on reputation for quality reporting with the intention of platform profit maximization in the IoT scenario. *RQRP* comprises two main phases: filtration in the selection of MWs and verifying the credibility of reported tasks. The former is focused on the selection of suitable MWs based on different criteria (e.g., reputation, bid, expected quality, and expected platform utility), while the latter is more concerned with the verification of sensing quality, evaluation of reputation score, and incentives. We developed a testbed to evaluate and analyze the datasets, and a simulation was performed for data collection scenario from smart sensing devices. Results proved the superiority of *RQRP* against its counterparts in terms of truthfulness, quality, and platform profit maximization. To the best of our knowledge, we are the first to study the impact of truthful reporting on platform utility.

## 1. Introduction

The Internet of things (IoT) is a broad concept involving a huge number of online smart devices that can communicate with other devices. Mobile phones, body sensors, GPS, gyroscopes, and a long list of gadgets are now omnipresent, being the lifeline of the modern world. IoT devices are greatly increasing in number every year, and 50 million are expected to be connected to the Internet by 2020 [1]. This drastic increase creates many challenges as well as opportunities. Trust in the provided services is expected to be one of the greatest issues of next-generation networks, where the social, cyber, and physical worlds of billions of IoT devices and humans will move side-by-side. Among these devices, the mobile phone may be the most influential and essential. The existence of cell phones has been exploited in many ways, such as in the paradigm of mobile crowd sensing (MCS) and mobile cloud computing [2]. Computation and sensing capabilities make it possible to lead MCS from wireless sensor networks (WSNs), due to their portability. Crowdsourcing is an emerging concept that brings opportunities by exploiting the abilities of crowd. Mobile crowd sourcing has also been exploited as a cloud service [2]. For the sensing task in MCS, mobile workers (MWs) have been employed. On the one hand, this is an advantage, but on the other, they may lack sensing quality. The closest concept to MCS is participatory sensing [3,4].

With the proliferation of mobile phone technology, mobile crowd sensing (MCS) is now a reality. The omnipresence of mobile devices presents a cheap way of getting services from the public at a distance [2]. Participatory sensing can be considered as a predecessor of MCS with unique implicit and explicit participation features. Data is collected from different sources (e.g., social networks and mobile sensing) by leveraging the intelligence of humans and machines together [5]. Need for the study of fusion patterns is identified that can help to integrate human and machine intelligence (HI and MI). An attractive comparison of wireless sensor networks (WSNs) and emerging MCS in terms of mobility, cost, and coverage is presented in [6]. This clearly shows the supremacy of MCS over fixed wireless sensors. Sensors in mobile devices can perform numerous sensing tasks (e.g., temperature, humidity, noise, etc.) with varying quality [7]. MCS is also extended to mobile crowd sensing as a Service (MCSaaS) [8]. A few well-known applications of MCS are traffic flow surveillance [9,10], noise [11], healthcare [12], and environment monitoring (urban monitoring) [13], where experiments are conducted on noise, air, and even electromagnetic fields as pollutants. A previous study [13] developed a suitable application to improve quality of life with the potential to help city planning authorities. Human involvement in MCS is beneficial, but also presents challenges such as ensuring the quality of sensing reports, privacy breaches, and maintaining consistent performance. Furthermore, attractive incentives are desired by participants. The sensing domain is categorized into participatory (conscious) and opportunistic (unconscious) sensing classes [14,15,16]. Hysense is a framework for MCS to compensate the uneven distribution of incentives by exploiting calibration, where MWs are instructed to move from areas with high population density to those that are less-densely populated [17]. A survey on the applications of MCS in industry and in the personal lives of common people was conducted by Shu et al. [18].

Several approaches to ensuring the quality of sensing in MCS have been proposed. Some of them are presented in the following. A skilled crowd with cheap services is a great advantage of MCS, but it can become a big drawback as well (e.g., when tasks can be submitted with false or undesirable quality). Selfish and strategic participants can act maliciously by delaying or manipulating the task completion properties to increase utility. Sensing reports can be malicious, or may be submitted to enjoy “free-riding”. Whatever the case may be, it costs platforms money to get reports from MWs [19]. To deal with the varying quality demands from task to task, Jiang et al. in [20] proposed a scheme known as the quality-aware incentive mechanism (QAIM), which should be efficient enough to correctly measure the report quality. Trust in the cloud environment is evaluated from the perspective of leaders influence based on different parameters in [21], and from requesters and crowd contributors in [22].

The main problem in MCS is that it is difficult to verify reported task quality due to the unavailability of ground truth in most cases. Even for situations where ground truth is available, the quality of current reporting cannot be fully judged based on previous records, as the current reporting may vary. Thus, platforms can be exploited easily and remain vulnerable to threats. There are three main entities in MCS: requesters, who are the consumers of collected data; the platform, which acts as a service provider; and MWs, who perform the sensing tasks. The selection of suitable MWs in MCS is one of the most important phases, which indeed is the first milestone. This can be a prior measure to ensure quality to some extent.

In the literature, data are collected and aggregated to get approximately correct results. In some approaches, aggregation was simply the average of the reported tasks, without the credibility of reporters and participation level, and rewards were paid irrespective of the contribution. Then, a weighted reputation approach was proposed in [23]. It also considered the issue of MW privacy, which is not the concern of the present work. Data perturbation is done by differential privacy-based crowd participation. With the intension to determine the quality of reported tasks from MWs, some approaches in the literature have used reputation-aware recruitment mechanisms. The IoT is an emerging paradigm, where reputation-based approaches [1] have been presented on the concept of collaboration. Game theoretic-based approaches have also been presented by considering the previously mentioned entities in MCS as game players, who are considered rational most of the time. The identification and correction of errors in reporting have gained the attention of researchers. Approaches which did not rely on history to inspect the credibility of sensing have also been put forward [24]. In contrast, we considered history as a helping hand for educated selection in order to achieve quality. Reporting quality remains a considerable issue, and several approaches have been proposed, but very few have considered reputation in this perspective. Some approaches have considered weight- and vote-based mechanisms, but are criticized for providing the right-of-vote to a few dominating entities and for not penalizing the malicious MWs. Cross-validation is proposed, which may require extra monetary incentives, and may not be suitable for budget-limited tasks. This raised the need to propose this work, so the existing gap may be filled.

In this paper, we present **R**eputation, **Q**uality aware **R**ecruitment for **P**latform (*RQRP*) to provide high-quality reporting in MCS. Reputation is one factor, and the inspection of credible reports is another. To achieve the desired quality, we divided our scheme into pre- and post-quality measures. For the pre-quality measure, we mainly considered reputation score and bids, and a few other task completion requirements such as time and lowest required quality are also considered. For the post-measure, we evaluated the quality after the sensing task was done and reported. We also considered feedback on the task from the requesters. To the best of our knowledge, we are the first to investigate the effect of un-trustable reporting on the platform profit in MCS. The main contributions of this paper are as follows:

(1) We designed a novel mechanism for mobile worker recruitment based on reputation level and expected quality of task. We present a recruitment mechanism to hire skilled MWs while mainly considering feasible budget, quality, platform utility, and individual rationality. In the similar vein, we propose a selection algorithm and reputation-updating system that considers the weight and score for both reporters and requesters.
(2) Next, we present a credibility inspection and incentive mechanism to reward or penalize MWs. We also present a novel algorithm for ensuring credible sensing. Additionally, our approach verifies the outcomes of MWs by considering sensing data from smart devices in that region for the IoT scenario. This helps to guard against false reporting from MWs and in taking strict actions in terms of penalties. For quality reporting, MWs are awarded. We are the first to analyze truthful reporting for platform maximization. The proposed mechanism is expected to ensure platform profitability with other task completion constraints while paying necessary incentives to the MWs.
(3) Finally, we developed a testbed using Windows Communication Foundation (WCF) services on Windows Azure cloud to evaluate and analyze the datasets containing MW reporting details. Moreover, we simulated the scenarios for collecting sensing data from smart devices and transmitting aggregated data at sink nodes via collectors. Sensing data are further saved in a database for analysis in combination with reporting data to identify false reporting by MWs. Results proved the dominance of our work as compared to its counterparts in the literature.

The rest of the paper is organized as follows: Section 2 presents related work in two sub-sections—incentive mechanisms and quality-centered approaches in MCS. Section 3 is devoted to the system model and problem statement. The proposed *RQRP*’s workflow and two phases are presented Section 4. Phase-A aims at the selection of suitable MWs in MCS by exploiting and enhancing the beta reputation system, which is also described as being the preliminary to the selection process. Phase-B is dedicated to evaluation of sensing reports for credibility along with updating reputation scores. Section 5 provides the theoretical analysis, and analysis of the results is presented in Section 6. In Section 7, we conclude our work and provide future work directions.

## 2. Related Work

The term “Mobile Crowd Sensing” (MCS) was coined in [4], and it has outstanding potential to exploit the power of crowds. Crowd Contributors (CCs) expect attractive reward for the contribution of their services. An efficient incentive mechanism is required to keep participants motivated to contribute remarkable sensing. MWs are selfish, so considerable efforts have been made in the literature to develop incentive mechanisms. We present some of these approaches to incentive mechanisms in this work. Due to the contribution from possibly untrustworthy participants, report quality is questionable. To come up with a solution, several approaches are presented in the domain of MCS. Various aspects have been considered on the behalf of the researchers by defining quality in different manners (e.g., low latency, small difference between ground truth and sensing reports). Reputation-based approaches also remain a point of consideration as a milestone toward the goal of quality sensing. There is clear evidence in the literature on the effectiveness of reputation-based approaches. Our proposed mechanism is concentric on reputation- and vote-based approaches. We explored state-of-the-art schemes, and critically analyzed these schemes to point out challenges and possible research directions.

### 2.1. Incentive Mechanisms in MCS

Crowdsourcing (CS) is based on outsourcing, which emerged with great potential in past two decades [25]. In any form, it provides the opportunity to deal with problems more effectively and efficiently. Two of the major divisions in mobile crowdsourcing are mobile crowd sensing (MCS) and mobile cloud computing (MCC) [2]. The emergence of wireless technologies was the foundation of MCS. Requesters, service providers, and workers are the key entities in MCS. Two mobile crowdsourcing architectures for MCS based on local- and Internet-level schemes are presented in [2]. Incentive mechanisms have been proposed in [19,26,27,28] to retain the interest of workers. Incentives can be paid by using: (1) auction and payment rules; (2) a lottery, where no perfect discrimination for the selection of winners is considered; or (3) trust and reputation, in which rewards are not monetary but can be a kind of social recognition or self-satisfaction [29]. In this work, we deal with monetary rewards only, which is more practical for study.

The mechanism designed in [24] did not utilize history for MW recruitment, opportunities in the MCS domain were explored and exploited efficiently. Control of MW selection was especially enhanced when crucial and important tasks were to be performed within budget constraints. The limitation of the work is that only homogeneous tasks were considered. The scheme in [30] presents two models: incentive mechanisms for crowdsensing systems under zero and general cases (IMC-Z and IMC-G). The zero model was designed when arrival and departure times were not considered. In contrast, the general model was presented where in–out time can be reported by MW. Observations were taken to set the benchmark for future recruitments, the scheme is focused on the cheap costs only, and truthfulness is considered. Another approach for truthfulness on the announced bids with the constraint of feasible budget is presented in [28]. In contrast to both of these approaches, we considered reputation as a quality insurance measure in the selection of MWs rather than least-bid criteria. The literature is also clear regarding the effectiveness of reputation-based approaches.

Lack of trust and quality on the work done by recruits have always existed in MCS. To ensure a trust level for accomplished tasks, platforms need to pay extra for the strategic and selfish agents in the form of more recruitments. MWs can perform maliciously, submitting false reports or inconsistent work, or it may be the case that an honest but curious worker delays the task for benefits. In this scenario, cross-validation can ensure quality but can be costly, so it is difficult for platforms to ensure a feasible budget [28,31]. A cross-validation scheme is presented in [32]. A unique feature of this scheme is that if a MW is not able to complete a task after being selected, another MW can be recommended by him. False reporting is one of the main issues causing lack of trust, so quality-aware truth estimation schemes are required [10,11].

Game theory-based approaches also remained a hot research area in MCS. The approach in [31] presented the problem of determining a budget with the assumption of perfect information. They proposed two incentive mechanisms for the CS environment: (1) frugal auction mechanism, which stimulates workers to report truthfully; (2) Stackelberg-game-based mechanism, where requester fixes the reward at the beginning and let the MWs to compete. Literature urge for robust evaluation scheme to guarantee the quality and creditability of aggregated task reports by avoiding MWs’ malicious behavior. Due to the threat of false and inconsistent task completion, the requester need to pay more than it deserves, which leads to the problem of budget feasibility [31]. Another prominent approach in the MCS paradigm that exploited Stackelberg-game and in which platform- and user-centric models are developed is presented in [33]. The objective of the platform model is to maximize the profit, whereas user model is aimed for the selection of time at which their utility can be maximized. A unique Nash Equilibrium (NE) is developed, and sensing time determination is handled in the user-centric model. A double-auction-based incentive mechanism for the case of multiple requesters which aggregates the collected data from users known as CENTURION was proposed in [34]. The mechanism also ensures various desirable properties of an efficient incentive mechanism. “Theseus” is proposed in [35], with the motivation of providing quality in MCS by stimulating workers to contribute accurate data to their best ability, and then data aggregation is done to ensure the quality, as in [33]. NE in a Bayesian setting is ensured in the proposed non-cooperative game in [36], while having individual rationality and feasible budget constraints fulfilled. Dynamic behavior is presented with evolutionary games, and competition among CSs is depicted by a non-cooperative game.

### 2.2. Quality-Centered Reputation-Based Approaches

The approaches in [37,38,39] present mechanisms which consider the quality of a task and the reputation of nodes in order to pay incentives. Gao et al. in [40] presented an approach which considers dynamic trust, wherein, direct and feedback trust are combined to hire well-suited MWs. Quality in sensing is a desirable property, which may require sufficient reporting to be ensured. This can be difficult with strict budget constraints. To deal with the problem of determining a feasible budget, the approaches in [28,31] are proposed. In [31], study is conducted on the extra payments which are just spent to introduce incentives upon job completion. Dynamic budget and quality in the MCS domain are presented in [41]. Restuccia et al. proposed FIDES in [42], which is an incentive mechanism framework designed to provide secure participatory sensing based on trust. They identified some threats for the incentive- and reputation-based approaches, and proposed threat models. To address collusion attack and to ensure credibility, FIDC is proposed in [43], which considers the correlation between spatial and sensing data with prior knowledge to avoid group-organized attacks (i.e., the injection of false data). By considering similar task requirements and users’ heterogeneous abilities, a three-layer approach is proposed with the aim of reusing similar data items. Task selection and user scheduling are jointly done with the purpose of increasing social welfare up to a certain level. Considering humans’ rating factors in mind, an approach is presented in [21], where requesters’ assigned quality is the benchmark of reward amount to the contributors. It presents a probabilistic model to quantify the error in assigning ratings, and ultimately its impact on incentives.

Pieces of art are proposed in [12,13,14,15] to obtain trustworthy work from employees. In [19], a scheme based on unsupervised learning for quality assurance on truthfulness is presented, where surplus is shared using the Shapley value as a cooperative game model. A reputation-based reward mechanism is used to obtain high-quality data in their approach for mobile crowdsensing. The use of the Shapley value provides a means of avoiding the free riding problem. To handle “free-riding”, a quality certificate was issued to the participants in [27]. This certificate can be used to monopolize the platform. In contrast to this, we used reputation to avoid free-riding that is also a kind of certification which provides the platform with expected contribution of MW, just we did not make it public to avoid the monopoly. This is useful, as it may be the case that constraints cannot be fulfilled without the contribution of some of the MWs, and thus those MWs can influence the recruitment.

Smart cities often have sensing activities to provide better services, for which they mostly rely on wireless communication. Smart mobile devices are used to contribute data at a large scale for the sensing of the smart city by dedicated or non-dedicated measures [44]. A recent approach aimed at green collaborative edge computing is presented in [45], where edge devices are installed to reduce the backhaul bandwidth. Another considerable effort was made in the development of an edge computing architecture for MCS application in [46]. A survey of the trust computation models for IoT systems and smart cities is conducted in [47]. Several aspects, such as trust composition, its aggregation, and its formation are calculated for privately owned devices (rented devices provide services only temporarily, and so can be used to act maliciously). Trust updation is done on event- and time-driven bases. The maintenance of trust for IoT devices can be centralized or distributed. The approach in [48] presented a research work by conducting a survey to achieve the quality of information (QoI) in the MCS paradigm. Several aspects have been pointed out as research challenges in validating the trustworthiness of QoI. A different approach is presented in [49] to ensure quality based on a contract. Crowdsourcing includes two kind of tasks: microtasks and macrotasks. A microtask does not require much expertise or time, and can be performed easily, but rewards are also low, whereas macrotasks are reciprocal of this. We considered microtasks in this work, which can be more challenging in ensuring the quality constraint in the presence of crowd participants. Microtasks in MCS have low MW utility most of the time, so it can be difficult to engage CCs in a contract. It can be useful for MWs’ and for the platform when MWs have fixed mobility patterns.

In [21], reliability is defined as the ratio of tasks completed globally and locally with defined weights given in Equation (1), where $\omega_{g}+\omega_{l}=1$:

(1) $$\;RE=\omega_{g}*\frac{Jobs\;Completed\;Globally}{Jobs\;Accepted\;Globally}+\omega_{l}*\frac{Jobs\;Completed\;Locally}{Jobs\;Accepted\;Locally}.$$

The reputation of individuals is calculated based on the number of accepted and completed jobs, and submitted tasks, and data integration, identity, and capability, as given in Equation (2), where $\omega_{1}+\omega_{2}+\omega_{3}+\omega_{4}+\omega_{5}=1$ are the weighting factors:

(2) $$R_{T}=\omega_{1}*\frac{Accepted\;Jobs}{Submitted\;Jobs}+\omega_{2}*\frac{Completed\;Jobs}{Accepted\;Jobs}+\omega_{3}*\frac{Data\;Integration}{Completed\;Jobs}+\omega_{4}*Identity+\omega_{5}*Capability.$$

Another way to get quality-oriented reporting is done by considering a collaborative approach rather than simple voting- or statistical-based trustworthiness, as in [50]. A similar approach presented collaborative trust in IoT based systems for the analysis of visitors’ behavior at a cultural event [1]. The proposed approach is attractive, as no single metric can influence at large scale, and it simply does not rely on the willingness of participants. A quality-oriented approach is presented in [51] for opportunistic networks, which may not be suitable for time-sensitive tasks.

Based on functional reputation, an approach in [52] considered the reliable aggregation and transmission of collected data by sensors in the WSN domain. It exploits “beta reputation” [53] to evaluate the trustworthiness of the node. In contrast, we utilized it in the MCS domain to ensure the trustworthiness of MWs. According to [53], a Bayesian estimation matrix can model mean based on a probability density function (PDF), and the prediction of credible contributions in the future from an MW can be made as presented in Equations (3) and (4). In our proposed *RQRP,* sensing reports can be accepted or rejected, as the nature of the beta system is binary. To differentiate between excellent, average, and normal contributors, we assigned ratings based on the individual and collective feedback from the requesters. Thus, the outcomes are partially binary in *RQRP*. From previous observations, the expected outcome for a new task can be expressed using ω=P+1 and γ=N+1, where P and N are positive and negative outcomes from total interactions, and estimation is given in Equation (4). In our approach, we took this concept and modelled it to estimate the reputation of the task at present using the history of previous tasks performed by the same MW.

(3) $$\;f\left(p|\omega ,\gamma \right)=\frac{\Gamma \left(\omega +\gamma \right)}{\Gamma \left(\omega \right)\Gamma \left(\gamma \right)}p^{\omega -1}{\left(1-p\right)}^{\gamma -1}$$

(4) $$E\left(p\right)=\frac{\omega }{\omega +\gamma +2}$$

The scheme in [39] covers various aspects, such as the availability and capability of a device, to analyze the trust in mobile phone sensing. Emphasis is made on the important role of reputation-based systems for MCS, as gadgets in this domain are owned by common people. Candidates’ reputation and weights are calculated as presented in Equations (5) and (6), respectively:

(5) $$R=\underset{i\epsilon s}{\sum }weights*\;R_{i,k-1},$$

where

(6) $$weights=\frac{R_{i,k-1}}{\sum_{i\epsilon s}R_{i,k-1}}.$$

In [39], the critique is given that beta reputation systems are not capable of penalizing malicious MWs for bad contributions. On the contrary, we adopted it by including penalty in terms of decrease in reputation and also by not selecting them as crowd participants. Furthermore, we employed a blacklist to punish malicious MWs. In our proposed work (*RQRP*), the trust mechanism based on a reputation system is installed in the platform as a central authority to maintain trust and sensing quality by assigning a trust score known as the R_Score. We also present a novel mechanism for rewarding MWs on the basis of task completion and score calculation. To the best of our knowledge, we are the first to study the impact of truthful reporting on platform utility with both parameters. Results showed that voting-based approaches were more prone to collusion attack. For approaches utilizing a majority voting concept, error propagates at a high rate. Most works which consider reputation-aware recruitments have counted on the probability of only those MWs with higher reputation. This means that experienced MWs will always have a greater chance of being selected, which may lead to monopolies based on reputation score. Voting-based approaches give the right-of-vote to only well-known entities, as in [54]. Whereas in our proposed approach, feedback on quality is not confined to fixed or predefined entities, and the credibility of the reports is also simultaneously considered.

Social aware crowdsourcing with reputation management (SACRM) [55] is presented with the idea of using social attributes for participants’ selection to perform sensing tasks. It measures the quality of the reported tasks in terms of expected and actual delay of sensing reports. Participation reputation is also maintained at the platform. Bonuses are paid to stimulate quality reporting. A limitation of the paper is that the platform is assumed to store the history of all the performed tasks and CCs, which can be impractical at large scale. A greedy approach was adopted in [56] to ensure quality while decreasing the sensing cost. Incremental reward is also considered by paying bonuses to the participants. Quality is measured by approximation ratio, but reputation is not considered. A unique approach with the aim of including quality in MCS by combining cyber-physical perspectives for geographic sensing is presented in [57]. For the selection of participants, simple aggregation of reports is exploited rather than reputation, and weightage is not considered, which is important for credible reporting. Recent work on the idea of profit maximization for MCS is proposed in [58]. Their main focus was same as ours, but they did not consider reputation as a benchmark for selection.

Our proposed work generates credible reports while being beneficial to the platform and fair to MWs. It is more generic than [31], as we considered mobile crowdsourcing which includes mobile cloud computing and sensing. We do not allow the MWs to compete for limited budget, and instead we involve multiple other parameters for MW selection. Moreover, *RQRP* can be applied on temporary bases at local mobile crowdsourcing infrastructures, as in [2]. We exploit reputation, whereas [28,30,32] did not consider it at all. The approach is also feasible for the online environment. Quality of sensing does not simply rely on the aggregation of reports, and for the sake of quality, no extra payments are made, which may occur in [32]. Budget was taken into consideration with dynamic properties, and the profit of the platform is given priority. On the other hand, the Designed Mechanism (DM) is expected to be IR (individual rational). In contrast to various approaches, we included two quality assurance checks: scrutiny of MWs and validation of reports. In contrast to the approaches which have used beta reputation, we also exploited an ageing factor, which may support the applicability of the DM at large scale.

Budget was divided into lower and upper limits for task completion, which can never be over-ruled. One of the reasons for setting a dynamic budget is to find and exploit the opportunistically available resources. Truthfulness is expected to exist, as there will be no benefit of false reporting. Payoff of the MWs is delivered depending upon the agreed-upon total value per task, contribution in big task, number of units performed (subtasks), and cost per unit, where quality of reported results is not neglected. The reputation updation system will influence MWs to work honestly, as this effects future hiring. This is in contrast to previous approaches, which utilized large crowds to take aggregate reports without making differences in weightage and thus needed more incentives, which may make it impossible to complete tasks with strict budget constraints. A unique feature that can help the platform to save storage is the concept of the ageing of history. Our scheme is efficient in this respect, as it requires less storage space and may have less running time for participant selection.

## 3. System Model and Problem Statement

The proposed MCS incentive mechanism *RQRP* model is presented in the following sub-section. The research problem, which is focused on achieving high quality of sensing while considering the social welfare of the participating entities, is also defined.

### 3.1. MCS Model

*RQRP* is defined as M(f,g), where f represents filtration and selection, and g stands for payments after the validation and reputation updation processes. The type of a MW is represented as $f\left(\theta \right)=\hat{\theta },$ where θ is the set of true types of MWs, and $\hat{\theta }$ is the declared type of MW as a function of f(θ). For the platform, θ is generated as function $f\left(R_{T},\;Q,\;S_{k}\right)$. In *RQRP*, $T=\left\{\tau_{1},\tau_{2},\tau_{3}\ldots {\;\tau }_{n}\right\}$ is the set of tasks, $U=\left\{u_{1},u_{2},u_{3}\ldots \;u_{n}\right\}$ is the set consisting of users, where n ε N={1,2,3…N}. After announcement of the task by the platform, MWs can bid on their cost. We assumed MWs to be the game-theoretic, so we considered $c^{\prime }=f\left(c\right)$, where c is true cost and $c^{\prime }$ is the declared cost. A MW’s bid is set as a triplet in our mechanism $b_{i}=\left(c^{\prime },q_{i},\;t_{i}\right)$, where i ε U, $c^{\prime }$ is the announced task completion cost, $q_{i}$ is the quality (which can be a function of skill and reputation), and $t_{i}$ represents the time in which $u_{i}$ can perform the task. In every case $t_{i}\leq d_{t}$, which is the time deadline. Time can be a function of distance between sensing and the MW’s current location. All of these are important considerations for the constrained aware selection of MWs. Collectively, for multiple tasks, cost cannot increase the budget, and reported quality less than the threshold is not acceptable.

The types of mobile workers can be categorized as: (1) honest MWs, which is the best case; (2) malicious MWs, who may deceive for incentives; (3) those who are not malicious but accidentally/infrequently report below expected quality; (4) those MWs who at first strategically contribute high-quality and then submit false reports and continue this to maintain trust above a certain level (known as an ON–OFF attack). Most of the literature ignores this kind of MW, whereas ON–OFF attacks can be handled by *RQRP*, as it is able to analyze the past behavior because history is maintained.

Skill level correlates with the ability to perform a task with desired quality. MWs with better skill level and lower bid are favorable to be the bid winners, and are more considerable if they are capable of performing multiple tasks. When a task is submitted, the DM ensures that the indifference between prior knowledge or instantly generated ground truth is not higher than expected. If so, then the task must be rejected. Ground truth is one of the important task quality factor. The objective is to validate the accuracy of the submitted task, especially when ground truth is generated from history. It is described as: if $\left[True\;knowledge\;–\;T_{i,j}\right]\;\geq \;\alpha$; then reject the submitted task, where α is a threshold parameter to investigate the quality, and in $T_{i,j}$ j is the submitted task by the user *i*. *RQRP* imposes a kind of filter to ensure the careful selection of MWs and the quality of reported tasks. The reputation score of an MW is one of the very important filters, alongside cost, expected quality, and MW skill.

Budget $\int_{B_{-i}}^{B^{+i}}B$ is set to be dynamic between lower and upper limits. The lower limit of budget is initialized with the rough estimate of the true cost from history, which can vary from task to task and is initialized by the platform. Different from most of the previous approaches, we considered that every bundle of tasks can have different sets of tasks with different budgets, which is a more realistic scenario. Two reasons to set a dynamic budget are as follows:

(1) Imperfect information about true cost of task completion of MWs.
(2) A variety of task completion requirements encourage dynamic budgets, as cost may vary from task to task with worker skill level, required quality, and with time sensitivity.

It is important to maintain the interest of MWs to get the task completed within the required constraints. Every MW expects attractive incentives, so an efficient incentive mechanism is most important for any efficient crowdsourcing platform—especially when decisions on the participation of workers cannot be reverted (e.g., online). Some important attributes that a good incentive mechanism should have are: truthfulness, if truth telling is a dominant strategy for MWs then the DM is truthful; individual rationality, meaning that at the least costs are paid to MWs; profitability, meaning that the DM should be profitable for the platform; feasibility—that is, if the task can be completed in polynomial time then the DM is computationally feasible; and fairness, which may hold if incentives are being paid according to contribution.

We assumed that MWs were aware about the presence of other MWs, who could also be the winner of the announced task, thus forming competition. The incentive of the upcoming task can be lower than already declared, and it may not be desirable for an MW to wait for the next task. All these factors motivate the MWs to bid on their true values, so we can expect that bidding based on the true cost will be the dominant strategy of MWs. This idea will lead MWs to perform at their best, irrespective of what other MWs’ bids are. As there will be no benefit of deviating individually, providing the best response will be desirable for the MWs. This concept is also known as the diminishing return in literature. The workflow of the proposed model is presented next. The most frequently used notations in this work are presented in Table 1.

**Figure 1:**
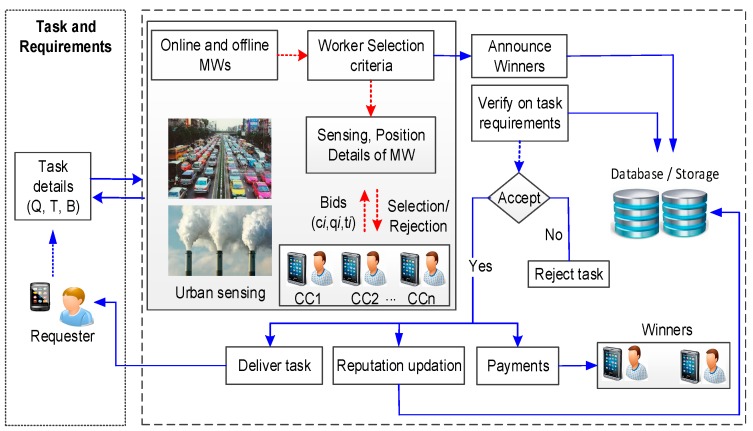
Proposed architecture of the Reputation, Quality-aware Recruitment for Platform (*RQRP*) method for task allocation and reward management. MW: mobile worker.

Figure 1 presents the designed *RQRP* architecture for MCS. It consists of two parts. In the first part, requesters declare tasks with required quality, budget, and time constraints. The second part is the most important part of the designed mechanism, and deals with the reputation-aware selection of the participants and the updation of reputation. Online and offline participants can both be handled, and worker selection based on reputation of task completion is the first milestone on the way to achieving high-quality sensing. To select suitable participants, the platform announces the task with constraints and waits for the MWs to bid, as handled by Algorithm 1 in Phase-A. We assumed the availability of enough MWs willing to participate in sensing tasks. When analyzing bids for the expected quality and platform utility that a particular applicant can provide, the mechanism selects one or up to the required number. After this, the platform announces the number of winners. From this point forward, Algorithm 2 (described in Phase-B) has a kind of interplay with Algorithm 1. Bid winners perform sensing tasks and submit the reports. The platform verifies the sensing reports with the task completion constraints and with the expectations from participants. Upon successful verification, payments are made to those winners whose tasks fulfilled the minimum criteria, otherwise the task is rejected. Rejection at this stage may encourage the worker to do their best for the next task. The platform delivers final report to the requester. On reply of requester for the contributed quality, reputation updation is performed. If the same task was required by multiple requesters, then reputation is updated by considering feedback collectively. Collective knowledge based on feedback is also useful to avoid the bias of requester opinions. Power to benchmark the quality is distributed and a final check is done at the platform. Reputation-aware recruitment allows us to conduct the selection of MWs to achieve the quality objective. Feedback from the requester can help the platform to predict recruitment. In contrast to some approaches which announce the reputation score, we did not do so, so that MWs cannot monopolize based on reputation score.

**Algorithm 1**: Selection of Suitable Mobile worker.**INPUT:** Attributes of task ($T,S_{k},\;Q,\;D_{t}),l\left(location\right),p\;\varepsilon \;P$, Assumption: Every MW has maximum task completion capacity $N_{ttc}\left[i\right]$
**OUTPUT:**
$N_{w},E\left(Q\right),E\left(c_{i}\right),\;E\left(P_{u}\right)$
   **1.** Initialize: $\left\{N_{w},E\left(Q\right),\;E\left(P_{u}\right),N_{cc},N_{tc}\right\}\leftarrow \left\{\emptyset ,Q,\emptyset ,\emptyset ,\emptyset \right\}$; $B_{-i},B^{+i}$  **2.** MWs(N) bids on their private value: $N_{CC}\left[i\right]\leftarrow b\left[i\right]$;  **3.** **For** (*i* = 1; *i* ≤ ($N_{cc}\left[i\right]$) && $N_{w}\;\&\&\;D_{t}\leq \frac{T}{2{\;}^{log}{}_{2}^{T}}$); *i*++)  **4.**  **If**
$N_{CC}\left[i\right]\geq (S_{k})$
**then**  **5.**   **If**
 E(Q)≥Q
**then**  **6.**    **If**
$\left(B_{-i}\leq b\left[i\right]\leq \;B^{+i}\right)$
**then**  **7.**     **If**
$(N_{tc}\leq N_{ttc}\left[i\right]$) **then**  **8.**       $N_{RC}\left[i\right]\left[i\right]$ //considered as real candidate  **9.**   **Else**  **10.**      $Reject\left[i\right]\leftarrow \;N_{CC}\left[i\right]$  **11.**  **End If**  **12.** **End For**  **13.** Sort list of $N_{RC}\left[i\right]$ in descending order w.r.t low bids b[i] and high $E\left(P_{u}\right)$  **14.** **For** any task **If**
$N_{RC}\left[i\right],\;b\left[i\right]\;\&\&\;E\left(P_{u}\right)$ are same **then**  **15.**  Select $N_{RC}\left[i\right]$ with higher R_Score or $R_{T}$  **16.**  Select the $N_{w}$ from the set of $N_{RC}\left[i\right]$ w.r.t Max $E\left(P_{u}\right)$, $N_{tc}++$  **17.** **End For**  **18.** Return $N_{w},E\left(Q\right),\;E\left(P_{u}\right),E\left(c_{i}\right)$

In Figure 1, the large dotted rectangle on the right side represents the methodology of our proposed *RQRP* as a whole. The dotted red arrows show the communication for mobile workers’ selection (Phase-A). This communication may contain the announcement of tasks, transmission of bids, reputation score, skill of the worker, or other requirements that must be ensured before the MW selection is made. These are the prior measures which set the ground for obtaining the desired sensing task quality, performed by Algorithm 1: selection of the suitable mobile worker. Blue arrows represent the credibility investigation, incentive assignments, updation of reputation into the database, and reply to the requester, which are mostly the objectives of credible sensing Algorithm 2 (Phase-B) of *RQRP*. 

### 3.2. Problem Definition

Based on the contributed quality of MWs from history, we created a skill matrix which is assumed to be the private knowledge of the platform. For every newly registered MW, the skill value in this matrix will be 0.5 by default but can vary depending upon the reported sensing quality. Skill level is defined as: $S_{k}=[\tau_{i,j}]\;\varepsilon \;{\left[0,1\right]}^{M*N},$ where τ is task, and i ε U, j ε T, and M∗N represent the columns and rows of the skill matrix. The reputation $R_{T}$ of any MW can be deduced from Q matrix, which is the contributed quality in history, whereas the Q matrix is the function of skill level $S_{K}$ matrix expectations before selection. Below, we present how the reputation is built from quality (feedback) and skill matrices:

$$R_{T}=\left[\begin{array}{ccc} R_{i,j} & R_{i,j\;}\ldots & R_{n,m}\\ R_{i,j} & R_{i,j\;}\ldots & R_{n,m}\\ R_{i,j} & R_{i,j\;}\ldots & R_{n,m} \end{array}\right]\overset{\;}{⇐}Q_{\;}\left[\begin{array}{ccc} Q_{i,j} & Q_{i,j}\;\ldots & Q_{n,m}\\ Q_{i,j} & Q_{i,j}\;\ldots \; & Q_{n,m}\\ Q_{i,j} & Q_{i,j}\;\ldots & Q_{n,m} \end{array}\right]\overset{\;}{⇐}S_{K}\left[\begin{array}{ccc} S_{i,j} & S_{i,j\;}\ldots & S_{n,m}\\ S_{i,j} & S_{i,j\;}\ldots & S_{n,m}\\ S_{i,j} & S_{i,j\;}\ldots & S_{n,m} \end{array}\right].$$

We defined our research problem in the following ways, presented in Equations (7) and (8):

(7) $$max\;Q_{t\leq \frac{T}{\left\lfloor 2{\;}^{log}{}_{2}^{T}\right\rfloor }\;}for\;l_{i}\varepsilon L\;\overset{N_{w}}{\underset{i\varepsilon RC}{\sum }}\;E\left(P_{u}\right),$$

(8) $$min\;t\leq \frac{T}{\left\lfloor 2{\;}^{log}{}_{2}^{T}\right\rfloor }for\;l_{i}\varepsilon L\overset{N_{w}}{\underset{i\varepsilon RC}{\sum }}\;E\left(c_{i}\right)\;\;as\;\;\overset{N_{w}}{\underset{i\varepsilon RC}{\sum }}\;E\left(c_{i}\right)\leq B^{+i}.$$

On receiving a bid from a crowd participant, the platform searches the matrices from the database to make a well-educated decision on the selection of mobile workers.

The objective of Equation (7) is to maximize the quality Q and expected platform utility $E\left(P_{u}\right)$ for any desired sensing location $l_{i}\varepsilon L$ until the $t\leq \frac{T}{\left\lfloor 2{\;}^{log}{}_{2}^{T}\right\rfloor }$, where T represents the deadline of performing the task, which is divided into slots. This is also the case with the budget $b\leq \frac{B}{\left\lfloor 2{\;}^{log}{}_{2}^{B}\right\rfloor }$, which is also dynamic as the time deadline varies accordingly, where B is the budget of all the tasks in one bundle. This division is the same as in [30]. Total task completion time is divided into slots and budget is set to be dynamic accordingly, which varies to meet the temporary deadline until Equations (7) and (8) are valid for $N_{RC}.$ $N_{RC\;}$ is the list of those MWs who are considered to be the real candidates because their trust scores are at least as high as the required quality for the sensing task. Selection of winning MWs $N_{w}$ is made until the time deadline reaches $\frac{T}{\left\lfloor 2{\;}^{log}{}_{2}^{T}\right\rfloor }$ $N_{w}$ is a set of winning candidates from the list of real candidates $N_{RC}$, who qualified in the first phase. For any declared sensing location $l_{i}\varepsilon L$, the objective is to maximize the expected platform utility $E\left(P_{u}\right)$ while ensuring the quality Q constraint until the deadline is reached. On the other hand, Equation (8) is aimed to minimize the cost of winning $N_{w}$ MWs which the platform is supposed to pay. It includes the sum of total costs of all the services offered by the set $N_{w}$. This must be done under the constraint that the sum of total expected costs $E\left(c_{i}\right)$ of every $N_{w}$ must not exceed the upper limit of budget $B^{+i}$, where $B^{+i}$ is the maximum cost that the platform can pay for the announced bundle of tasks.

**Algorithm 2**: Credible Sensing.
**INPUT:**
$G_{t},\;l_{i}\;\varepsilon \;L,,S_{r}\left[i\right],Q\left[i\right],p\;\varepsilon \;P$

**OUTPUT:**
$W_{u},\;N_{w}\;Q_{Score}/R_{T},\;p$
   **1.** $\mathrm{Initialize}:\;N_{w}\left(S_{r}\right)\leftarrow \mathrm{Accept}\left(1\right)/\mathrm{Reject}\left(0\right),\;Q\_Score\leftarrow 0$  **2.** **For**
(i=0; i ≤
*T*; *i*++)  **3.**  **If**
$S_{r}\left[i\right]\;of\;h(l_{i})\;\varepsilon \;H(l_{i})$] **then**  **4.**   **If**
$\left(S_{r}\left[i\right]-h(l_{i})\leq \beta \;\&\&\;E\left(Q\right)\geq \alpha \;\&\&\;S_{r}\left[d_{t}\right]\leq D_{t}\right)$
**then**  **5.**    **If**
$Q\_Score=\left|\;E\left(Q\right)-R_{q}\left[t_{i}\right]\;\right|>Q\left[i\right]$
**then**  **6.**      Accept $S_{r}\left[i\right]\leftarrow N_{w}\left[i\right]$  **7.**       $R_{T}=R_{T-1}+\beta R_{T-1}$   // increase in reputation  **8.**   **Else**  **9.**     $RejectS_{r}\left[i\right]\leftarrow N_{w}\left[i\right]$   // add the MW’s task in rejected array of $RejectS_{r}$  **10.**    $R_{T}=R_{T-1}-\beta R_{T-1}$   // decrease in reputation as penalty  **11.**   **End If**  **12.**  **End If**  **13.** **End For**  **14.** **For**
$\mathrm{any}\;S_{r}\left[i\right]\notin h(l_{i})\;but\;Q\geq \alpha$  **15.**  $W_{u}=p$  **16.**  $h(l_{i})\leftarrow S_{r}\left[i\right]$  **17.**  $R_{T}=R_{T-1}+\beta R_{T-1}$  **18.**  $\mathrm{Assign}\;\mathrm{weight}\;S_{r}\left[i\right]$ according to *R_Score*;  **19.**  **If**
$S_{r}\left[i\right]$ is reported by newly recruited MW **then**  **20.**    *R_Score* is initialized by 0.5;  **21.**  **End If**  **22.** **End For**  **23.** $\mathrm{Return}\;N_{w},N_{w}\left(R_{T}\right)$   // winnersandtheir quality scores  **24.** $\beta =\sum \left(S_{r}\left[i\right]-h(l_{i}\right))/Total\;S_{r})$   // β is updated for upcoming task to set benchmark

The social welfare of the system is also considered in our DM. Social welfare is a term borrowed from microeconomics, and has the goal of participants’ satisfaction. The DM for MCS is the interplay of three common entities: MW, platform, and requester. In some studies, the platform and requester are considered as one entity, but we considered them as two separate agents. The DM in our proposed work is a system that ensures the satisfaction of all three parties. Social satisfaction can be defined as the net profit for all entities. In our scenario, we defined the social welfare as in Equation (9):

(9) $$S_{w}=\overset{N_{w}}{\underset{i=1}{\sum }}\;P_{u}+\overset{N_{w}}{\underset{i=1}{\sum }}\;\left(p_{i}-\;c_{i}^{\prime }\right)+\overset{Req_{\;}}{\underset{i=1}{\sum }}\;Q_{i}.$$

In Equation (9), Max $\sum_{i=1}^{N_{w}}P_{u}\geq C{,}_{\;}\mathrm{where}$ C represents the total cost borne by the platform, and its utility will be greater than or equal to C. This is ensured by the selection filter in *RQRP*, where no combination of MWs will be selected in which C will be greater than the upper limit of the budget $B^{+i}$. The DM is also profitable for the MWs, as $p_{i}-c_{i}^{\prime }$ will be at least greater than or equal to $c_{i},$ which is the true cost of the MW. This property can also be satisfied by the individual rationality attribute of DM, which means that in general scenarios, MWs must be paid their declared cost $c_{i}^{\prime }$. On the other hand, we defined the satisfaction of the requester in terms of achieving the quality $S_{r}\geq q_{i}$, where $S_{r}$ represents the sensing report and $q_{i}$ is the threshold value of task acceptance.

## 4. Proposed Reputation Quality Aware Recruitment for Platform (RQRP)

**Figure 2:**
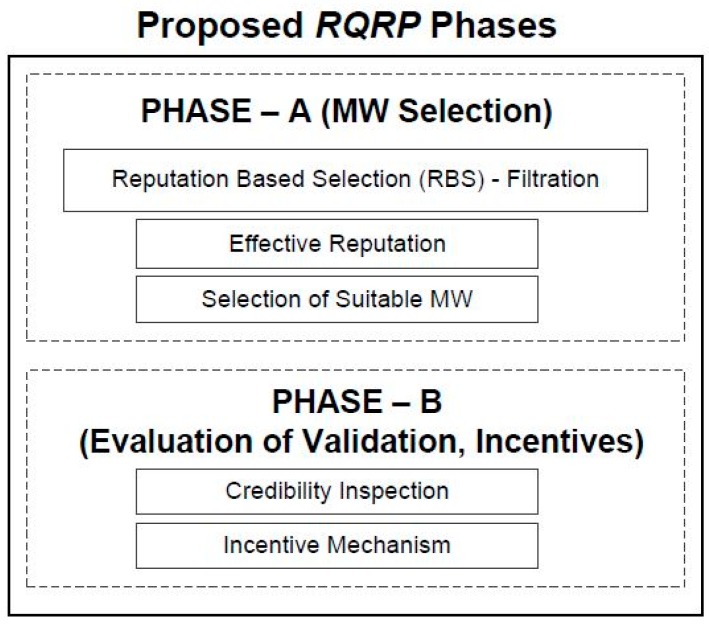
Phases of proposed *RQRP* for MW selection (**top**) and evaluating validation and incentives (**bottom**).

The DM is distributed into two phases, as shown in Figure 2. Phase-A has two sub phases: filtration and the recruitment of suitable MW(s). Phase-B also has two stages: credibility inspection of reports and assignments of incentives to the platform and MWs. To design a mechanism that can maximize the platform utility while ensuring the quality of reporting requires that different challenging tasks be confronted, enumerated as follows:

(1) Selection of suitable MWs by fulfilling the task’s constraints.
(2) Validation of task quality is necessary, as MWs can submit low-quality reports and may want to enjoy a free ride. They can also be selfish, strategic, and may intentionally manipulate results to misguide the platform. To avoid all this, quite a strict check and balance should be maintained on submitted reports. The challenge lies in how to ensure the quality of reports.
(3) Enforcement of work quality. The development of an efficient system which can hire trustworthy CCs is necessary. Furthermore, there should be a method to avoid the monopoly of MWs, which is also a necessary step to maintain quality by keeping their interest.
(4) Ensuring that budget and time constraints are operated within.
(5) Stimulation of MWs with a proper incentive mechanism, which can handle online mobile crowdsensing task distribution.

**Figure 3:**
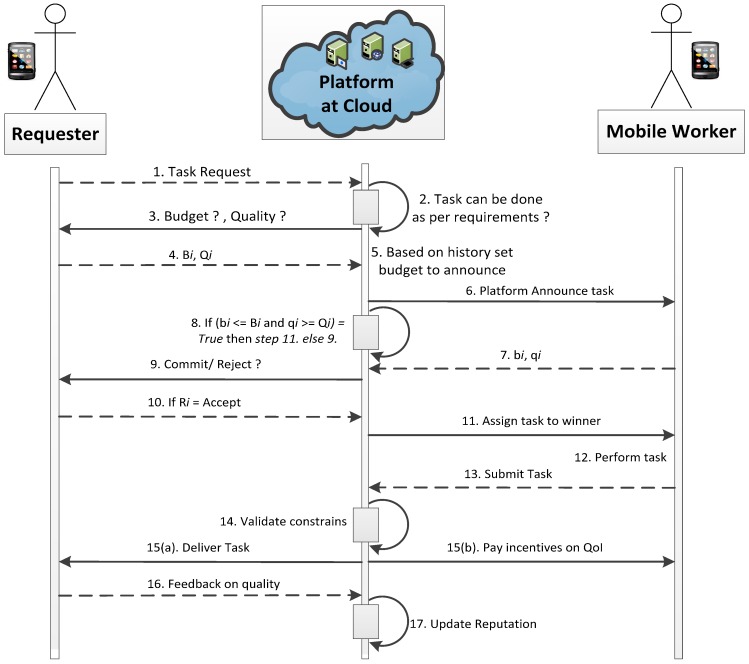
Details of work flow for quality-aware mobile crowd sensing (MCS) in *RQRP*. QoI: quality of information.

Sensing in MCS may include images, videos, temperature measurement, environment monitoring, and much more. The proposed *RQRP* aims to achieve quality of sensing based on reputation, where incentives are paid on the contribution. Its application is not limited to one scenario, and can be utilized for any of the previously mentioned application examples, where CCs/MWs need to be recruited, quality of reporting should be ensured, and incentives are given in reward of services. Due to its foundation in the “beta reputation system”, it is also presented after necessary enhancement to adapt to the MCS environment. Different from the available literature, our designed approach is more suitable for the following reasons: (1) It can manage offline and online scenarios at the same time, where users dynamically join and leave. In this situation, decisions are made in real time; (2) *RQRP* creates competition among MWs to have continuous effort, whereas most approaches in the literature only emphasize reducing the cost of hiring; (3) Reputation-aware recruitment provides the chance for the selection of suitable MWs with enhanced trustable quality reporting. It also discourages false reporting, which was totally ignored in [30,36,59]. (4) The feasible budget constraint is considered while being profitable to the platform. Necessary payments are made to keep the interest of MWs. (5) Truthfulness is expected to be achieved, as it is the dominant strategy for the players to bid on the true value. We assumed that platform would have some prior knowledge about the true costs of MWs. This estimate can be obtained from previously completed tasks. To ease the reader’s flow, we present the sequence diagram of *RQRP* in Figure 3.

On task generation from the requester with the details of task requirements, platform take some necessary steps, it may consists of multiple servers as shown in Figure 3. The platform checks whether the task can be accomplished or not. The server at the platform can be a certificate server which maintains authentication services, or it can be a database server that can store the history of task completion for the participants at large scale. Our proposed scheme is a bit more flexible than some of the approaches in the literature, as it does not simply deny the task request due to constraints on it. Rather, based on history, it can negotiate with the requester on quality with the declared budget before announcing the task to the MWs. After the task announcement, MWs submit bids. If the task cannot be completed within required quality and budget limits, then the platform can inform the requester with the changing state in step 9. This kind of situation can occur as an assumption is made on imperfect information, so states can vary, even after consultation of history. Otherwise, the platform selects winners based on $b_{i\;}\leq \;B_{i}$ and $q_{i\;}\leq Q_{i\;}$. After task completion, the MW submits their report then the platform analyzes the task and delivers it as shown in step 15(a), only if basic constraints on the task are fulfilled. At the same time, a reward based on contribution quality is assigned in step 15(b). Participants’ reputation is also updated to enable well-informed recruitment decisions in the future based on the feedback of requester/requesters as shown in step 17.

In *RQRP*, we assumed that MWs are game-theoretic. Thus, the probability of false reporting or selfish behavior by MWs does exist. We considered the malicious responses, whereas for the MWs with long-term history of job completion with the platform are less expected to be malicious. One of the novel features of our approach is that we maintain a blacklist of MWs. This list contains the malicious MWs, but it needs to be made carefully, as it can be the case that a mobile worker is not malicious but submitted a low-quality task while having a good prior reputation of job completion. Situations like this can sometimes happen, for a number of reasons (e.g., environmental factors). If a mobile worker is consistently submitting poor-quality reports and their R_Score (reputation score) drops beyond a certain level, then this MW can be considered as malicious, and should not be recruited. 

### 4.1. Phase-A: MW Selection

Phase-A of *RQRP* involves the selection of MWs who can maximize the utility of platform while ensuring the required quality standard is met. Careful selection of CCs can ensure budget feasibility, with no extra money to validate the sensing reports. In the next sub-section, we present our reputation-based selection, which is actually a filtration process. This stage is important for the goal of quality.

#### 4.1.1. Reputation-Based Selection (RBS)—Filtration

Reputation-based selection (RBS) consists of initial filtration and selection. In general, we assumed that the platform had a history of previous tasks, and on the arrival of any new task, the platform announces it with all the details except for budget in order to create competition. Until response from MWs, the DM finds the list of matching MWs with the required skill for the announced task. After that, upon receiving bids, the DM compares the attributes sent by the MWs with those MWs who had completed tasks successfully in the past and who are also currently in a position to perform the recently announced task. After this, the DM can select the most suitable MWs to fulfil the task completion requirements. This is the initial filtration process. We dedicated Algorithm 1 to the selection of suitable MWs and explain the process hereafter. In contrast to the available literature, we assumed that there is a maximum task completion capacity. For the MW selection, we consider the trade-off between service quality and true MW cost. However, the true cost of the MW is unknown in most cases, and so it is considered as incomplete information as mentioned previously. Rough initial information of task completion can be derived by the platform’s previous recruitments for the same kind of task with reported quality. This is done by beta reputation [53], described in the following.

#### 4.1.2. Effective Reputation

We considered effective reputation in two aspects. The first is the direct feedback from single/multiple requesters on the MW’s sensing task. Reputation from multiple requesters should be considered if the MW was recruited and completed multiple tasks. The second is the platform’s own trust calculation for the MW based on historic observations. We considered this because remarks from requesters can be biased due to human factor (liking or disliking) or the requester’s own skill in evaluation. Thus, it is logical that we should consider the platform’s own opinion for the reputation score (R_Score) of the MW as a whole. The “beta reputation system” is an effective approach used for the calculation of trust level. For effective reputation, we also considered an ageing factor. The purpose of using this factor is to reduce the impact of prior reputation scores on the current MW selection. It seems to be realistic that previous R_Scores should not be considered forever, as the performance of CCs may vary from time-to-time depending on the situation. Even though the performance of a specific MW does not change in their local scenario, there can be other new employees who can do a better job, so the ageing factor is useful. On the other hand, the ageing concept ultimately reduces the impact of history to zero after a certain time. Two other aspects are worthy of consideration: the first one is that real-time response can be obtained by reducing the recruitment selection time. The second is that storage space can be saved, allowing more records to be maintained. We used the term of “weightage” for requester and “reputation” for the MW. We discuss the reputation of the MW, the weightage of requesting, and updating records in the following.

##### (1) Reputation of Mobile Worker

Reputation calculation and updation involves various aspects, and some important equations and their description are presented. Requester feedback is key to the reputation procedure. Other than this, we do not just give the right-of-vote to a single requester, especially when there are multiple requesters who have requested same task. Equation (10) explores *R_T_*, which is the reputation of an MW based on the ratings assigned by the requesters. $W_{i}$ represents the weightage of the requester’s feedback and $R_{k}^{T}$ is the reputation score as a whole.

(10) $$R_{T}=W_{1}R_{1}^{T}+\;W_{2}R_{2}^{T}+\ldots \;W_{N}R_{N}^{T}=\overset{N}{\underset{K=1\;}{\sum }}W_{K}R_{K}^{T}$$

##### (2) Weighting of Requester Rating

The proposed *RQRP* is unique from other reputation-based approaches, as we do not maintain the reputation of only MWs. We also tried to analyze the ratings given by the requesters on sensing tasks, so that human bias can be removed and an efficient reputation-based mechanism can be designed for MCS. $W_{1},W_{2},\;\ldots \;W_{N}$ are the weightage of the requesters for the case of multiple tasks, if the requester is assigning ratings honestly and it is not drastically different from the ratings assigned by other requesters on collective bases. The weighting capability of that requester’s given rating will increase, and otherwise decrease in the same fashion. Weight is calculated from the given weights in history, and is simply the average of *n* previous weights as $W_{T}=\sum_{K=1}^{n}\frac{W_{n-k}}{n}$.

##### (3) Weight Updation

Weightage given to the MWs must be updated to analyze their contribution with the passage of time. It should also be updated because the platform should not reply on all of the past contributions for every selection. Thus, if the rating given by the requester/requesters is within the standard deviation of the ratings given by multiple requesters, it means that a particular requester is assigning a true rating in correspondence with other requesters, so their rating weightage weight will increase accordingly as shown in Equation (11) where $U_{R}-\sigma_{R}<R<U_{R}+\sigma_{R}$. Here, C is a constant factor and N is the number of tasks in records to be stored in history. Factor N can be adjusted by the platform, where a large value of N means more history has to be traversed in order to calculate the rating weightage. Similarly, if the rating assigned by a requester is not within the standard deviation, then the rating weightage decreases when $U_{R}-\sigma_{R}>R>U_{R}+\sigma_{R}.$

(11) $$W_{T}=\overset{N}{\underset{K=1}{\sum }}\frac{W_{T-k}}{N}+C\overset{N}{\underset{K=1}{\sum }}\frac{W_{T-k}}{N}$$

(12) $$W_{T}=\overset{N}{\underset{K=1}{\sum }}\frac{W_{T-k}}{N}-C\overset{N}{\underset{K=1}{\sum }}\frac{W_{T-k}}{N}$$

##### (4) Task Rating

We measured the rating for single and multiple tasks. The rating for a single task is given in Equation (10), where *W_N_* represents the weights of rating given by the requester and $R_{N}^{T}$ is the rating given by the requester for any task T. Thus, the rating for a single task done by the MW is $R=\sum_{K=1}^{n}W_{N}R_{N}^{T}$. The average rating of *M* tasks is taken as $R=\;\frac{1}{M}\left[R_{1},\;R_{2}+\ldots +R_{M}\right]$. By combining rating of multiple tasks, we get Equation (13):

(13) $$R==\frac{1}{M}\left[\overset{N}{\underset{k=1}{\sum }}W_{N}R_{N}^{T_{1}}+\overset{N}{\underset{k=1}{\sum }}W_{N}R_{N}^{T_{2}}+\ldots +\overset{N}{\underset{k=1}{\sum }}W_{M}R_{M}^{T_{M}}\right]=\;\frac{1}{M}\left[\overset{N}{\underset{k=1}{\sum }}\overset{M}{\underset{j=1}{\sum }}W_{J}R_{J}^{T_{k}}\right],$$

where *R* is the rating calculated for the tasks that are currently performed. Overall reputation depends on the current rating and the rating from history, which may have different weights in Equation (14) where $h_{1},\;h_{2}\ldots \;h_{N}$ are the weightages of previous task ratings stored in the history such that $h_{1}>h_{2}>h_{3}\ldots >h_{N}.$ Rating weights decrease while moving back in history and eventually become zero. From that point on there is no need to store the history, which also saves storage space for the platform. On the other hand, it brings a decrease in the computation time, because less history needs to be traversed and $R^{T-k}$ is the corresponding rating. Equation (14) represents the reputation for a single task, whereas Equation (15) represents reputation by combing multiple tasks:

(14) $$R=h_{1}R^{T}+h_{2}R^{T-2}+\ldots +h_{N}R^{T-N}=\;\overset{N}{\underset{L=1}{\sum }}h_{k}R^{T-k},$$

(15) $$R=h_{1}\overset{n}{\underset{K=1}{\sum }}W_{1}R_{1}^{T-n}+h_{2}\overset{N}{\underset{K=1}{\sum }}W_{2}R_{2}^{T-k}+\ldots +h_{m}\overset{n}{\underset{K=1}{\sum }}W_{n}R_{n}^{T-k}=\overset{m}{\underset{K=1}{\sum }}\overset{n}{\underset{L=1}{\sum }}\;h_{k}\;W_{L}R_{L}^{T}.$$

To eliminate/reduce the requester’s feedback bias, we considered collective feedback, especially when similar tasks are requested by a number of requesters. For such a scenario, we used the standard deviation by setting it to the aggregate of the feedbacks from different requesters. For the filtration phase, we developed some criteria to select a set of winners from candidates as below. Reputation is not the only parameter of selection, whereas budget on the collective bases should also not be violated along the many others mentioned prior.

$$\{\begin{array}{l} if\;any\prod_{i=1}^{n}C_{i}\;of\;\left(R_{N_{C}}\right)>\;B^{+i};\;then\;Reject\;candidates\\ if\;any\prod_{i=1}^{n}C_{i}\;of\;\left(R_{N_{C}}\right)=B_{-i};\;then\;Accept\;\left(preferred\right)\;\\ if\;any\prod_{i=1}^{n}C_{i}\;of\;\left(R_{N_{C}}\right)\;>\left(B_{-i}\right)\;but\leq B^{+i};\;then\;Accept \end{array}$$

The second combination is the most preferred situation. If it comes true then there is no need to go for a third possible combination of participant selection. These criteria simply check the combination of costs that need to be paid in order to recruit MWs. Before selecting any one or a set of MWs, the system quantifies the expectations (quality, cost, etc.) of the platform for any MW who is bidding against any task ($T_{i}\;or\;subtasks\;=\left\{t_{1},t_{2,}t_{3}\ldots t_{n}\right\}$) that could possibly be assigned to the winner. Expectations in view of previous performance records are defined as:

(1) For any ${\mathrm{MW}}_{i}$, if her R_Score is highest among $N_{cc}$ (crowd contributor), it ensures the task completion requirements will be met, and no other currently available online MW with a better offer than this MW is likely to be selected.
(2) The DM computes the probability of expected quality based on R_Score of $N_{RC}$ (real candidate). Any candidate with higher probability has a higher chance of selection.

#### 4.1.3. Selection of Suitable MW

For Algorithm 1, we assumed that every MW has maximum task completion capacity $N_{ttc}\left[i\right]$, and there are enough MWs willing to perform the task. Now, we describe its worker selection procedure whenever a task and its details are announced and MWs make bids in response. We assumed that MWs bid on their private value (dominant strategy) while being aware of the presence of other mobile workers and the strong quality evaluation procedure at the platform. Inputs to the algorithm are ($T,S_{k},\;Q,\;D_{t},),l\left(location\right),p\;\varepsilon \;P,$ where T is the task, $S_{k}$ is the required skill level, Q represents the required quality, $D_{t}$ is the deadline to perform the task, l is the sensing spot, and p stands for the maximum payment as reward for task completion. We assumed that every new worker who is willing to be assigned the tasks must be registered with the platform first. By doing so, a history of recruitments can be maintained. This does not mean that the DM just handles the offline working environment. Any new worker is welcomed to perform task and gets incentives as reward after finishing sensing task successfully. By giving the opportunity of selection to the new MWs, we can remove the monopoly of old MWs who are already registered and have high R_Score. An important assumption is that the platform maintains a history of workers. The value of the expected quality can be E(Q)=[0−1], which is measured before the recruitment of a MW. If history does not exist then we set it as (0.5) by default, similar to the case with $S_{k}$. If a bidder has history at the platform, then expected quality and expected platform utility are measured as:

$$E\left(Q\right)=\frac{Quality\;of\;Reported\;Tasks\;}{No.\;of\;Tasks\;Performed},\;E\left(P_{u}\right)=\frac{h(P_{u}-R(N_{W}))\;}{Total\;Tasks\;of\;Similar\;Nature}.$$

These two are two important attributes in the selection of MWs. A higher value of expected quality for task completion with optimum profit is the desired situation for the platform.

The DM sorts the R_Scores of the bidding MWs in descending order. As is clear from Figure 3, upon receiving a task from the requester, the platform searches for any available MWs who can perform the sensing task by satisfying the task completion constraints. After completion of this search, the DM compares recently found candidate MWs with the list of MWs in historical recruitments who are capable of performing tasks similar to the announced task. For any candidate MWs ($N_{cc}$) matching with the historical recruits, their R_Score can play a role in the selection. The point to be considered here is that if the DM only relies on the R_Score, then the MWs can influence the selection procedure. In this case, we can say that the DM is biased in some way. To mitigate this bias, we add sensing capabilities as a prominent feature, which means that much better quality can be obtained with better-skilled MWs. Due to this, a newly registered MW can take part in sensing tasks. The objective of these efforts is to get truthful and authentic sensing reports from MWs by denying the possibility of monopoly. Thus, there is always a competitive environment for MWs, which can be help to maximize the platform utility and obtain good quality reporting as well. The R_Score can vary between [0–1]. This parameter is a kind of task completion probability for a worker with given constraints. The selection of well-reputed MWs is the key to get better-quality sensing tasks. A parameter (alpha) is set to make a threshold for the selection of MWs.

Step 3 of Algorithm 1 is the iteration criteria until online candidates are available to be selected and the deadline is not yet reached. Steps 4–7 verify the fulfilment of task constraints with respect to different parameters. For any $N_{CC}$ crowd contributor, E(Q) is the expected quality based on previous reputation from that CC, b[i] is the bid of the MW and $B^{+i}$ is the upper budget limit that should not be crossed accumulatively. Total task completion capacity is presented by $N_{ttc}\left[i\right],$ where $N_{tc}$ is the number of currently assigned tasks to a MW. If a bid is not based on a true valuation of the MW or is beyond the task’s expected value to the platform (higher than the upper budget bound), the DM will reject the MW’s bid. Especially, when the platform has a history of task assignment available as a benchmark, as well as reward and quality scores of reported tasks, then the selection decision can be much more educated. The set of candidates who are able to complete announced task from the list of CCs are presented are presented as $N_{RC},\;N_{CC}$ respectively. Step 13 sorts bids with respect to platform utility and expected quality. For the MWs whose bids and platform utility are the same, then selection will be decided based on the greater expected quality value by iteration of the “FOR” loop in steps 14–17. R_Score and $R_{T}$ are used interchangeably, and so should be considered as the same until mentioned otherwise. In step 16, the selection of the winner is done based on platform profitability from the array of real candidates. Finally, step 18 returns the number of winners, expected platform utility, expected quality, and expected cost to be paid to the MW in reward of service.

### 4.2. Phase-B: Evaluation of Validation and Incentives

Phase-B of our proposed work is designed to evaluate the credibility of the reported sensing tasks by the participants. The criteria of acceptance or rejection at the platform for the MW’s report are based on the minimum acceptable quality of submitted tasks. If a task is rejected, there may be reasons other than the required quality. During the evaluation, one possible cause of rejection can be late submission. The platform validates/verifies task completion quality with the available ground truth from history. In case ground truth is not available, then currently submitted results can serve on its behalf by taking an average, so an acceptance/rejection decision can still be made. Later, this initial ground truth can be analyzed and updated to provide a better benchmark of quality. In this way, the platform can deal with any task requested for the first time by the requesters which has not been sensed before. New ground truth can be considered and refined. After validation is done, an incentives mechanism is presented to assign the utilities of MWs and the platform.

#### 4.2.1. Credibility Inspection

Sensing credibility is very important in MCS, as the participants are from the crowd and thus can be unreliable. Our work is different from some of the approaches in literature who rejected the task and did not pay any incentive to MWs. In our approach, once an MW is short-listed for task completion, it means that at least the initial criteria are fulfilled. For that particular MW, even if the sensing report is rejected, the platform can still pay only to those MWs who meet the minimum reputation qualification. This payment can be made as some ratio of the bid. However, at the same time, reputation will be degraded as a penalty and if there is a gradual decrease the DM will automatically move that MW to the blacklist. On one hand, it looks like a loss for the platform, but it is necessary as a selected MW should not be discouraged because bad sensing reports can be generated even from well-reputed MWs. This is also necessary due to the importance of incentive mechanisms in MCS.

Now, we can move further to elaborate the working of Algorithm 2, which aims to evaluate the credibility of submitted sensing reports. The DM calculates but does not announce the R_Score to the workers, as the MWs may exploit it to emphasize their selection. In our DM, the R_Score is a private value of the platform for any MW who bids on an announced task. The R_Score is of key importance, as it can create a competitive environment among MWs and can also stimulate them to produce high-quality work to the best of their abilities and resources. A better R_Score means a greater chance of selection as a winner for the tasks to be announced in future. Algorithm 1: “Selection of suitable mobile worker” is designed to select the suitable MW based on various constraints, such as task completion quality and budget constraints. In making the selection decision, reputation is also taken into consideration as mentioned in Equations (10)–(15) as per requirements. Once recruitment is completed and sensing reports are submitted to the platform, Algorithm 2: “Credible sensing” inspects the quality of sensing reports based on task completion criteria. Other than this, participant reputation is also updated and incentives are paid. Thus, we can say that the output of Algorithm 1 is the input of Algorithm 2. Next, we explain the working of Algorithm 2.

Steps 1–13: Initialization is done from the database when the ground truth for the announced sensing locations is available. If the ground truth cannot be deduced from currently available history, then it is considered as a new sensing location. Other inputs of the algorithm are: $S_{r}\left[i\right]$—sensing report from MW[i], Q[i]—the required quality for any task T[i], and p ε P—the payment that is expected to be paid taken from Algorithm 1. For the received report of a task, if the history for that sensing location does exist, then a comparison can be performed and the decision of acceptance or rejection can be made easily. If the difference of the reported task is less than or equal to the β threshold parameter (the minimum acceptance criteria deduced from history), the expected quality is greater than the α (a range of expected quality for a MW) and the task submission deadline is not already passed, then task is acceptable. Otherwise, it is rejected in steps 4 and 5. If the task is acceptable, then the algorithm checks for a quality score as well, $R_{q}\left[t_{i}\right]$ is the real reported quality of a report, $N_{w}\left[i\right]$ is added to the array $AcceptS_{r}\left[i\right]$ (the array of accepted sensing reports) and $R_{T}$ is reputation, which is updated (increased) in step 7. If $S_{r}\left[i\right]$ is rejected, reputation updation still must be performed accordingly by decreasing the reputation score for that MW as presented in step 10. If an MW’s sensing task is rejected again and again and their reputation is dropped below a certain level, then that MW can be moved to the blacklist. This is a unique feature of *RQRP* which can save platform assets from malicious MWs based on repetitive rejection. This process is done repeatedly until all the sensing reports are benchmarked for which history is available and incentives are also assigned accordingly.

Steps 14–22 deal with the case where history is not available to set the ground truth for the reported task. In such cases, in order to quantify the sensing reports for task quality factor, we assign the weights according to the R_Score of the reporter to set the ground truth for future reporting. Meanwhile, payment is made and reputation is updated according to $R_{T}$. In this perspective, our designed approach is different from most in the literature which simply set the ground truth by taking the average of reported tasks. Finally, the algorithm returns the updated R_Score of the MW and updates the list of $N_{w}\left[i\right]$. By this mechanism, any advertised sensing task can be performed which was never sensed before by setting the reputation and enhancing the trust level. Parameter β is updated in step 24 for well-predicted future recruitments.

To avoid the complexity of handling reputation from multiple requesters, the algorithm is presented for the simple case of single-task reputation updation. More general scenarios can be handled in accordance with Section 4.1.2, which discusses reputation-based selection. For example, when multiple task scores from history are taken, feedback from the requesters can be the aggregation of all of the positive and negative feedback against the reported task.

The total cost paid to the MW is a function of unit cost, which can also vary from task to task, depending upon the constraints. These constraints can be on quality, skill level, deadline of task completion, and the number of successfully completed tasks by all the winners. We assumed that cost is paid on the base of each bundle so can vary and also by considering the number of tasks performed with respect to the unit cost of each task. Cost paid on the all the tasks collectively is presented as $C_{n}=\left[(\tau_{i}\right)\left(c_{i}^{\prime }\right)]$, where $c_{i}^{\prime }\varepsilon \;C$. $\tau_{i}$ is the task and $c_{i}^{\prime }$ is the cost to be paid on completion of any task.

#### 4.2.2. Incentive Mechanism

Once the output of the selection algorithm is produced, winners are announced, and tasks are reported and validated for contributed quality. Now, we present how the payments should be made upon the successful completion of tasks. Those MWs whose bids are not accepted are not incentivized. The incentive of MW may vary from time to time, even for the same task, depending upon task completion constraints like quality, total number of tasks, units performed, and cost.

#### 4.2.3. Utility of Platform

The utility of the platform can be calculated by subtracting all the payments made to the MWs from the total gained profit. Payments are calculated as below by Equation (16), where $N_{w}\;\varepsilon \;N$ and $n\;\varepsilon \;N_{w}$ which stands only for those MWs who are going to be paid by the platform. As the main objective of this work is to fetch the quality of sensing reported from common people who can be more uncertain, we paid attention to this point and payments are also made depending on the quality of contribution. That is why P represents the payment as a function of Q, which is the quality of the reported task.

(16) $$P_{u}=\overset{N_{w}}{\underset{i=1}{\sum }}Pf\left(Q\right)-\overset{N_{w}}{\underset{n=1}{\sum }}C_{n}$$

#### 4.2.4. Utility of MW

The utility of a MW n where $n\;\varepsilon \;N_{w}$ is calculated by Equation (17), where $p_{i}\;\varepsilon \;P_{\;}$ is the total payment made to the MW on successful completion of task/tasks, and the second part represents the total true cost of the MW, which is a private value.

(17) $$W_{u}=\overset{n}{\underset{i=0}{\sum }}p_{i}-\overset{n}{\underset{i=1}{\sum }}c_{i}$$

## 5. Theoretical Analysis

Analyses of desirable DM properties are presented here, such as truthfulness, platform profitability, individual rationality, polynomial time computation, social welfare, feasible budget achievement, and fair dealing.

### 5.1. Truthfulness

MWs are selfish and strategic, and so want to maximize their reward. Meanwhile, truthfulness is the best-case scenario. The designed mechanism is truthful if truth telling is the best response strategy of a CC and users have no benefit from unilateral deviation—any bid beyond the upper limit of the platform budget will definitely be rejected. In this way, the feasible budget constraint is also fulfilled. If participants decide to take part in the sensing task, then it will be beneficial for them. For example, when MWs have defined mobility patterns, it will aid them in obtaining some revenue. Equation (18) indicates that the first derivative gives the positive maximum reward and the second derivative is negative. This proves that it will be the dominant strategy for a MW to bid on the true cost, as the reward cannot be increased further. Truth telling will also be the dominant strategy, because the platform will not pay beyond the budget limit and the reward for upcoming tasks may be less than the currently announced one. This is also known as “diminishing return” in [31]. Worker utility $W_{u}$ is a strictly concave function. It will increase with the sensing time and growth rate (i.e., first derivative is positive whereas second derivative is negative, which means the maximum achievable utility from the given function and cannot be raised further by manipulating the strategy.)

(18) $$\frac{dW_{u}\;}{d\tau_{ij}}\geq 0\;and\;\frac{d^{2}W_{u}\;}{d\tau_{ij}^{2}}\leq 0$$

### 5.2. Platform Profitability

The designed approach is profitable, especially in the sense that the same kind of multiple task completion requests may be requested for sensing. An application scenario for this could be a grand gathering of a crowd for an event, which may require the sensing of road traffic, air pollution (PM2.5), noise pollution, etc. On the other hand, as the selection criteria are also based on various parameters, the extra incentives paid elsewhere in the literature can be saved, especially for the random selection-based mechanisms. 

### 5.3. Individual Rationality

Payment to an MW is at least as great as her bid for successful completion of the task, and it must be made based on the true cost or on the type of MW. The agreed value must be paid by the platform after evaluation of the reported task in light of the feedback from the requester, individually or collectively. Payment is made based on contributions like the number of units performed, cost per unit, and quality. MW’s reward at the least should not be less than the rough estimate of his true cost. The MW’s utility can be calculated as $W_{u}=\sum_{i=0}^{n}p_{i}-\sum_{i=1}^{n}c_{i}\geq MW_{tc}$. If this condition is satisfied, then it means that the utility of the MW greater than or equal to her total true cost $MW_{tc}$. Then, we can say that the DM is individually rational, as at least the true cost will be paid. Furthermore, when an MW is selected based on bid and the required quality of task completion, he will be paid by some ratio of the bid, even if the sensing report is rejected. In this special case, individual rationality will be redefined such that the incentive will not be paid as the full bid. A decrease in reputation will also be made as a penalty, which will boost the dedication to quality sensing for the next task.

### 5.4. Time Computation

**Figure 4:**
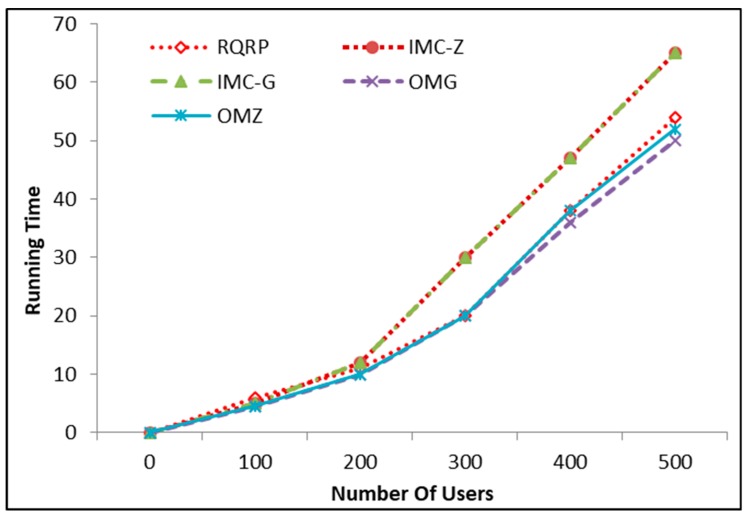
Comparison of running time with some of the approaches from the literature. IMC-G: incentive mechanisms for crowdsensing systems under general case; IMC-Z: incentive mechanisms for crowdsensing systems under zero case.

Even after including the traverse of reputations from history at the platform, the DM is computationally feasible, as indicated by the results in Figure 4. Algorithm 1 is devoted to the selection of suitable mobile workers, and Algorithm 2 is responsible for the credibility inspection and reputation updation. In Algorithm 1, step 3–12 perform MW selection, which is dependent on the number of users, and compares their n bids. Thus, time complexity is O($n^{2}$). Step 13 is the sorting process, and so has the time complexity O(*n* * (log *n*)). Steps 14–17 have the time complexity *n*. Thus, the overall time complexity of Algorithm 1 is O($n^{2}$). In Algorithm 2, the first step is the evaluation of submitted sensing reports, which has constant time complexity as per the number of reports. Step 2–13, step 14 and step 22 assign updated reputation values and so have constant time. The overall time complexity of Algorithm 2 is constant (*C*) according to the submitted reports to inspect the credibility. Figure 4 shows that the running time was almost similar to contemporary approaches.

## 6. Results and Analysis

In this section, we present the results to evaluate the performance of our proposed *RQRP* in comparison with the based schemes. We set up a testbed by implementing WCF services using C# and ASP.net to deploy on the Windows Azure cloud. We adopted responsive design to enforce the provision for visibility of our application on mobiles, tablets, and laptops regarding screen scaling. We maintained records in the SQL Server database for evaluating the recruitment and credibility of reporters by calling ADO.net APIs along with Language Integrated Queries (LINQs) using Lambda expressions. Moreover, we evaluated the Gawalla and T-drive data sets, which contain check-ins performed by users in different locations in California, USA and taxi GPS traces in Beijing, China, respectively. In California state, every check-in user willingly declared his location information including latitude and longitude, resulting in better tracking and analysis. Additionally, we also simulated the proposed *RQRP* using NS 2.35 to perform the data collection mechanism from sensors on smart devices in the IoT scenario. We have developed separate C files to differentiate the sent, received functionalities of low power sensing devices and high power data collectors Sink nodes. Next, we extracted the data from the sink and incorporated it in a separate table of a SQL Server database to evaluate it in conjunction with MWs’ reporting data to identify false reporting scenarios. Whenever a task is announced and winners are selected through Algorithm 1 to perform the sensing task, the selection procedure exploits the R_Score, skill level, bid, expected quality, and utility of the platform. The platform’s utility and sensing task quality are the two most important concerns of our work.

Evaluation parameters are provided in Table 2, along with the range of values utilized in the results and analysis. Initially, the R_Score of a participant was 0.5. The minimum expected R_Score was based on the task quality factor α as 0.3, 0.4, 0.5 for the selection of MWs. Later, based on the contribution of MWs, the reputation score varied (increases or decreases), representing the contributed quality. An increase in R_Score value is a kind of guarantee of quality sensing. The values of α can be viewed as the required submission quality for a task that must be fulfilled by the bidding MW to be selected for task accomplishment and to get incentives. Expected reward and availability of competent crowd contributors are inversely proportional, and vice versa. We consider the user’s check-in as the completion of the sensing task. We dealt with a more realistic scenario by considering the probability of successful task completion after getting bids and making the selection of suitable MWs. Commitment level of task completion can also be computed for a selected MW who was recruited in the past. Its value can indicate current task completion probability. This can lead to well-guided selection, especially when MW availability is not an issue. $Cb=\left(TN_{w}-Ttask\right)/TN_{w}$ can be used to calculate the intactness (commitment) of an MW to task completion. $TN_{w}$ is the number of selected winners, which can be considered up to a specific time from history. Ttask stands for total task announced, and it can be calculated for MWs individually. Results proved that our proposed reputation-based approach outperformed its counterparts. Results on running time, platform utility, truthfulness, impact of reputation, and quality of reporting are presented next.

The considered datasets include details of MWs and tasks in a large area of 1000 m × 1000 m. To present the running time, we changed the number of users from 100 to 500 and the number of tasks from 100 to 300, where the mobility of each user is taken as 30 m, which means MWs are only considered as CCs when they are in a 30 m radius of the generated sensing task. The task completion capacity of each user is taken as 1, 5, and 10. To show the effect of ageing factor on the storage capacity, we changed the ageing factor from 0.3 to 0.5.

### 6.1. Running Time

The running time of the proposed *RQRP* is presented in comparison with other approaches in Figure 4. It showed a linear increase with the increase in the number of MWs. We considered the reputation of MWs, which requires time to traverse the record for educated selection. Still, the running time of all of the schemes was almost the same. To decrease the traversing time, we have exploited the concept of ageing. 

This reduced the size of effective history to achieve low latency. Late history is removed with the passage of time, so that newly updated reputation is considered for selection. This also saves storage space at the platform and has potential for application at large scale. Typically, *RQRP* took 11 ms for 200 users, which is 8% better than IMC-Z [30] and IMC-G [30] approaches, which took 12 ms for the same number of users, whereas OMG [28] and OMZ [28] took 10 ms. Thus, even after considering the reputation, *RQRP* was in competition while ensuring quality.

### 6.2. Platform Utility

**Figure 5:**
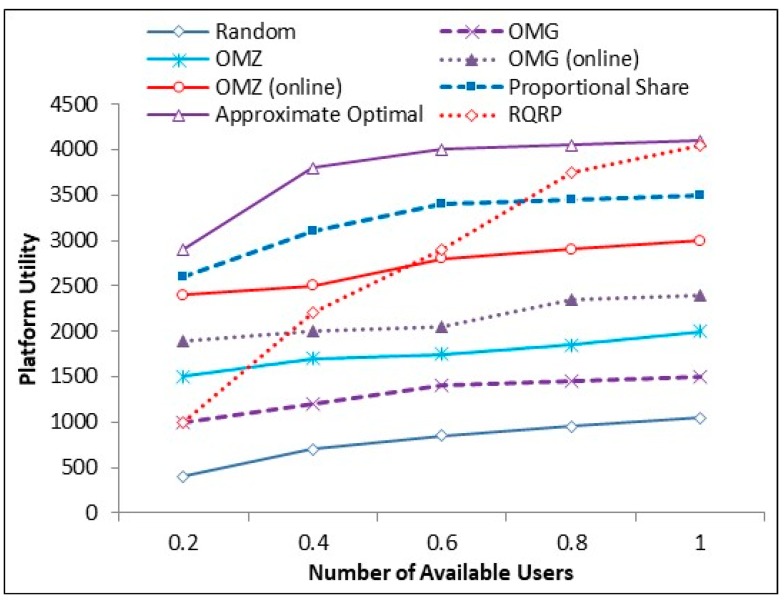
Effect of change in the number of MWs on the platform utility.

Approaches are compared with respect to the platform utility in Figure 5. *RQRP* showed a gradual increase as the trust level on the MWs is increased with the passage of time. The reason for this continuous change is that reputation is only considered in our approach. When the number of MWs is increased, the platform’s utility showed an increasing trend. Consideration of reputation created a competitive environment and the platform needed to pay less. If reputation is not considered more MWs may need to be recruited, which requires monetary incentives. In contrast, *RQRP* rejected many candidates based on low reputation, which also saved the platform’s resources. An MW whose score was less than 0.3 from the maximum value of 1 was rejected as the general criteria. The change (increase/decrease) in the score value is dependent on the contribution made. Platform utility increases directly with the rate of available users, as more options are available. For example, on the arrival rate of 0.6 users, the platform utility for RQRP was 2900, whereas 700, 1200, 1700, 2000, 2500, 3100, and 3800 were the utility values for random, OMG [28], OMZ [28], OMG (online) [30], OMZ (online) [30], proportional share, and approximate optimal approaches, respectively. We present the results in large integral values in order to the meet with the scale in the approaches compared.

We selected the IMC, OMG, and OMZ approaches for the comparison because: (i) these are some of the well-known state-of-the-art approaches in the MCS paradigm; (ii) these approaches have similar input/output constraints to ours; (iii) in contrast, these approaches lack the use of a reputation-based mechanism, which could have played a promising role in increasing the quality of sensing in MCS. These schemes are based on the idea of taking samples first then making acceptance, which is somewhat similar to our proposed work. The very basic difference is that we considered reputation when making a selection decision at first stage, and a lower bid was not the only selection criterion.

### 6.3. Truthfulness

**Figure 6:**
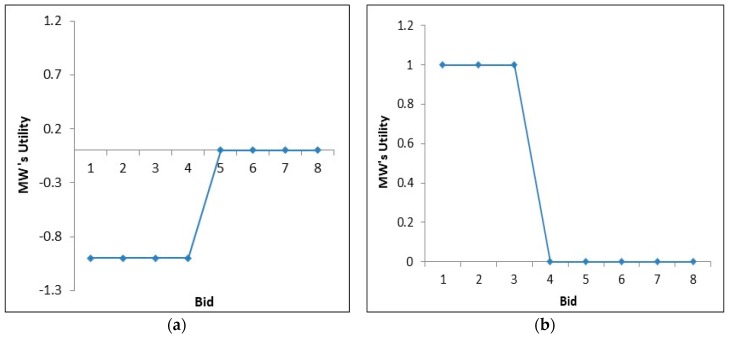
Cost truthfulness for (**a**) T-drive dataset and (**b**) Gowalla dataset.

Figure 6a,b represent the truthfulness on the T-drive and Gowalla datasets, respectively. The figures illustrate the impact of truthful announcement of cost on the utility of MWs. If MWs report cost untruthfully, they may not get any reward, making it beneficial for crowd contributors to bid on true cost. The platform will not pay any combination of costs greater than the budget, so truthfulness can be achieved in *RQRP*.

### 6.4. Platform vs. Mobile Worker Utility

**Figure 7:**
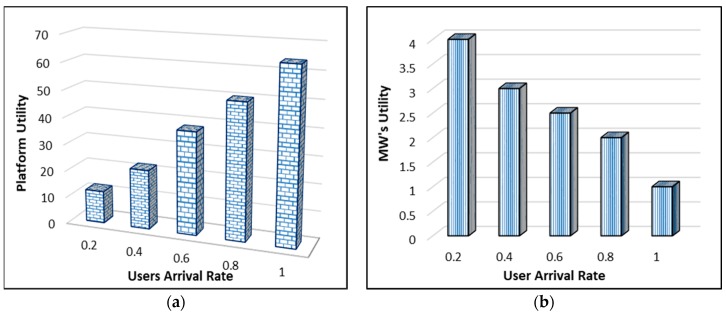
Users arrival rate compared with utility for (**a**) Platform and (**b**) MWs.

The impact on the utility of the platform and the MW with respect to the online available MWs was also analyzed. Figure 7a shows that utility of the platform increased with increasing number of online MWs. This increase was due to the large sample size of mobile participants and the competition among them. Due to this competitive environment, the platform needed to pay less and its marginal utility was increased. On the other hand, the utility of the MWs showed a decreasing trend with the increase in number of participants in Figure 7b. This is because of the declared fixed budget to be distributed among MWs. Our DM is individually rational (IR), as MWs are paid their costs. Thus, in the end, the MWs will not regret contributing. An increase in the available online participants had a gradually increasing impact as far as the utility of the platform was concerned.

This effect had an inverse impact on the MWs’ utility. The increasing trend is due to the richness of participants with on-board sensors within mobile gadgets. As the number of MWs increases, their utility is expected to be decrease, so the ratio is inversely proportional. This effect is the same as in [27]. In Figure 7b for the simulation, we took the average of 100 values. The reason for having similar values for both cases could be that users were selected at similar bid values and sometimes may be at higher or lower values. However, the similarity of values could be attributable to the fact that we took the average of 100 values.

### 6.5. Required Quality vs. Quality Delivered

**Figure 8:**
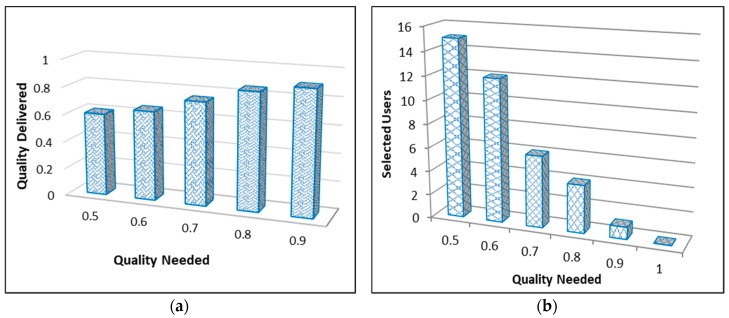
Quality needed is presented for (**a**) delivered quality and (**b**) number of selected users.

Figure 8a presents the needed quality in comparison with delivered quality on task completion by the proposed *RQRP*. The figure shows that there was slow but gradual increase until the maximum quality (one) was required. Users were not selected until the reputation constraints were met. If a user is selected, he most probably contributes the sensing task with similar or higher quality to his previously contributed quality. Thus, on average, delivered quality always is greater than required quality. Tasks are not accepted until the constraints are fulfilled, so incentives are not paid. For example, in Figure 8a for the quality constraint of 0.6, the delivered quality was 0.65, which is higher than the required quality. In MCS, it is very difficult to achieve doubtless quality due to the presence of various participating factors (e.g., hardware installed, experience of MW, intention of the MW to participate in sensing task).

### 6.6. Required Quality vs. Selected MW

Figure 8b shows the number of selected users in comparison with the required quality. Acquiring 100% quality in the MCS domain is challenging, as the mobile devices are owned by common people who can be vulnerable, malicious, and may lack in experience. As our DM’s basic objective is to achieve quality, Figure 8b represents the needed quality of the tasks on the x-axis and the number of selected users to obtain the desired quality on y-axis. We noticed that with the increase in the required quality, fewer MWs were selected. For the case when average quality 0.5 from the maximum possible contribution of 1 was considered, more users could contribute to the sensing task/tasks. A quality of 1 is almost impossible to achieve because this means that there should not be any difference between ground truth and the sensing report submitted by the MW. With the increase in required quality to the maximum possible, the figure showed that it was possible for there to be no user able to make any contribution. For example, for the required quality of 0.7, the number of selected users was 8, whereas for the quality of 0.8, the number of selected users decreased to 4. This is due to the increase in quality constraint leading to fewer users qualified the task completion criteria.

### 6.7. Impact of Quality on Reputation

**Figure 9:**
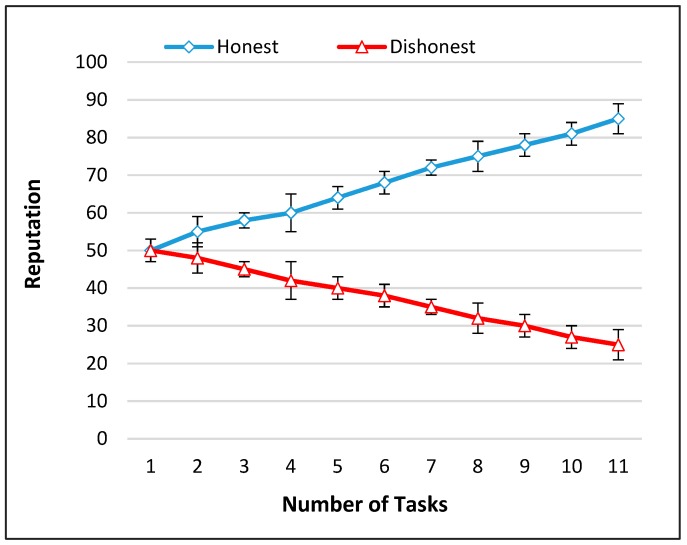
Reputation of honest vs dishonest MWs.

Quality of the sensing reports is ensured based on the various measures. At first, we considered reputation as a prior measure and then inspection of credibility as a second step. Figure 9 shows the change due to honest and dishonest MWs. For the honest MWs, there was increase in the reputation, which ultimately recommends them for future selection. Well-reputed MWs can be a symbol of surety for the better expected quality of tasks. Whereas, reputation decreased for the dishonest MWs, as shown. If a MW continuously reports low-quality sensing, he will eventually be deregistered, and can also be added to the blacklist. Decrease in reputation is a kind of punishment. By penalizing the MWs for their bad contribution, we also tackled the criticism of reputation-based systems in MCS.

### 6.8. User Reputation 

**Figure 10:**
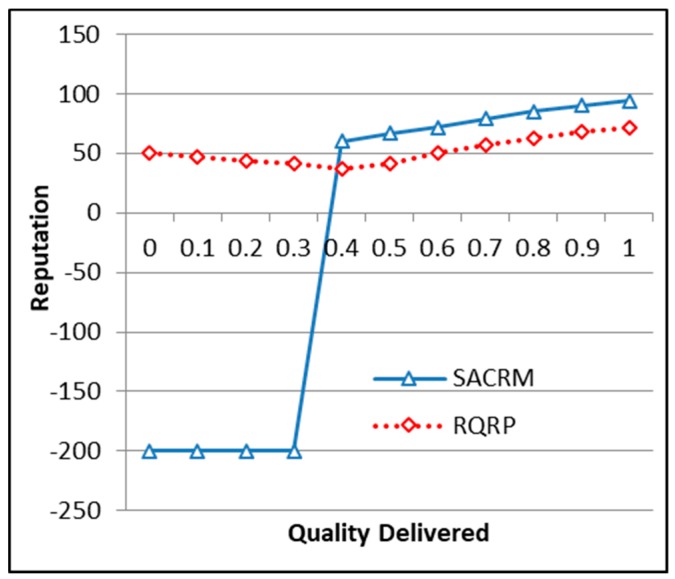
Delivered quality vs. reputation score. SACRM: social aware crowdsourcing with reputation management.

For simulation purposes, we used values in percentage for number of attributes and for reputation [0–100] as presented in Figure 9, which actually may range from [0–1]. This is the analysis of our proposed approach only. Whereas, when comparison with SACRM was required, we compared it by using integer values as shown in Figure 10. This represents the effect of reputation with the change in quality reported by the MWs. The similarity between SACRM [55] and *RQRP* is that both approaches consider reputation. An exemplary application scenario may be that when a few MWs are required to perform a task that is requested by multiple requesters, the platform can deal with this situation on a whole bundle basis, which can increase the profit of the platform. A limitation of this work is that although the DM makes expectations of quality based on reputation for task completion, sometimes expectation can go wrong as the MW’s task completion capabilities may vary from time to time. This is one of the uncertain situations that can arise even after the careful selection of MWs, and even after the exploitation of an efficient reputation updating mechanism.

### 6.9. Error Bars

**Figure 11:**
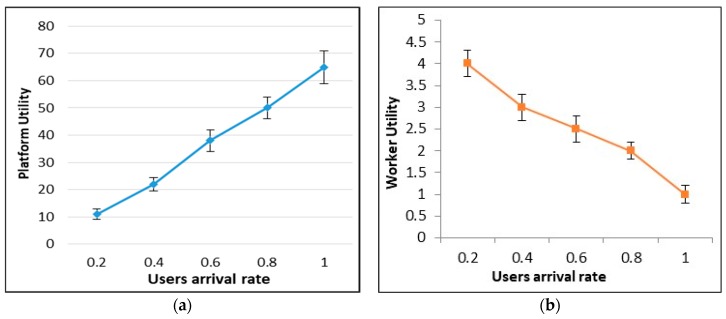
Users arrival rate for (**a**) platform utility and (**b**) worker’s utility.

Error bars are presented below in Figure 11a,b to show the deviation from mean values as the experimental results may not always be precise. The confidence levels of the simulation are presented by sampling errors in Figure 11. Estimation of platform utility with their deviation from the mean is presented in Figure 11a, whereas Figure 11b presents the estimation of mobile workers’ utility with their deviation from the average value. Average estimated value of utility was considered, and deviation from the average value over the iterations are presented with error bars. Larger sample sizes may have small differences, whereas small samples may vary largely.

## 7. Conclusions

The IoT brings opportunities and challenges at the same time. Most approaches in the literature for MCS are lacking as they do not include reputation in their consideration, which can be one of the reasons for low-quality sensing. This can be a cause of untrusted MW selection without any specific criterion, and ultimately extra monetary incentives are wasted to increase the approximate quality of reporting and the utility of the platform is ignored. We proposed the *RQRP* mechanism for MCS, which considers reputation as an important aspect in the selection of MWs to ensure the quality of reports and platform utility in the presence of malicious and selfish MWs. The proposed approach is broadly divided into two phases: (i) selection (ii) validation and reputation updation. Selection is made carefully, as we assumed that in most cases ground truth is not available to compare the quality of reports, which is a more crucial and realistic scenario for task accomplishment. The validation and reputation updation phase helps to verify the reports and to maintain the reputation of MWs for future hiring. An ageing factor is used to reduce the impact of past reputation score. *RQRP* is suitable for offline and online MCS environments. Simulation results proved the superiority of the proposed approach. The DM ensured truthfulness, computational efficiency, individual rationality, and most importantly the profitability of the platform with the required quality constraints. For user arrival rate of 0.6, our technique provided 30%, 40%, 50%, and 70% more platform utility than OMG (online), OMZ, OMG, and random techniques, respectively. For future research direction, a privacy preservation-based approach shall be proposed to deal with MWs’ security. Moreover, the MCS framework can be proposed for vehicular networks with a variety of sensors that increase coverage, which was limited in the case of mobile phone users.

## Figures and Tables

**Table 1:** Most frequently used notations in this work.

Notations	Description
R_Score	Reputation score
$P_{u},\;W_{u}$	Utility of platform and mobile worker
$E\left(Q\right),\;E\left(P_{u}\right)$	E(Q)> is expected quality from bidding mobile worker and $E\left(P_{u}\right)$ is the expected platform utility
$T,t_{i\;}or\;\tau_{i}\varepsilon T$	Task, Subtasks
$D_{t}$, $G_{t}$	Deadline of task completion, and ground truth
$Q,R_{q}\left[t_{i}\right],Q\_Score$	Desired quality of task, $R_{q}\left[t_{i}\right]$ is the real reported quality of any task according to ($G_{t},R\_Score$), and Q_Score is quality score after task completion
$\alpha ,\beta ,b\left[i\right],\;b_{ij}$	α,β are threshold parameters, b[i] is the bid of any mobile worker ${\mathrm{MW}}_{i},$ and $b_{ij}$ is the bid of any ${\mathrm{MW}}_{i}$ for task *j*
$S_{k}$	Expected skill level
$N,\;N_{c},\;N_{RC},N_{w},N_{tc},N_{ttc}$	N is the total number of mobile workers, $N_{c}$ is the set of candidates who have submitted bid*s,* $N_{RC}$ is set of candidates who are considered as real candidates, $N_{w}$ is the number of winning MWs, $N_{tc}$ is the total task assigned, $N_{ttc}$ is total task completion capacity of MW
$S_{r},G_{t},l_{i}\;\varepsilon \;L$	$S_{r}$ is sensing report, $G_{t}$ is ground truth, $l_{i}\;\varepsilon \;L$ is a sensing location from a set of locations
$B^{+i}$, $B_{-i}$	Upper and lower upper limits budget
$c_{i}$, $C_{i}$	$c_{i}$ is the unit cost paid to the MW whereas $C_{i}$ is the total cost paid to one $MW\varepsilon \;N_{w}$

**Table 2:** Parameters for the evaluation criteria of *RQRP* and its counterparts.

Parameter	Value
Target area	1000 m × 1000 m
Number of MWs	100–500
Tasks announced	100, 200, 300
$N_{ttc}$	1, 5, 10
Least task quality factor (α)	0.3
Effective mobility region	30 m
Reputation score	[0–1]
Default reputation value	0.5
Ageing factor	0.3–0.5
